# Aldosterone and Its Blockade: A Cardiovascular and Renal Perspective

**DOI:** 10.1100/tsw.2006.68

**Published:** 2006-04-03

**Authors:** V. Lahera, V. Cachofeiro, G. Balfagon, J.L. Rodicio

**Affiliations:** ^1^ Department of Physiology, School of Medicine, Universidad Complutense, Madrid, Spain; ^2^ Department of Physiology, School of Medicine, Universidad Autónoma, Madrid, Spain; ^3^ Department of Medicine, School of Medicine, Universidad Complutense, Madrid, Spain

**Keywords:** aldosterone, mineralocorticoid receptor antagonists, cardiac alterations, vascular disease, renal damage

## Abstract

Aldosterone not only contributes to salt and water homeostasis, but also exerts direct cardiovascular and renal effects. Numerous experimental and clinical studies indicate that aldosterone participate in cardiac alterations associated with hypertension, heart failure, diabetes and other pathological entities. It is important to mention that dietary salt is a key factor in aldosterone-mediated cardiovascular damage, since damage was moreevident in animals on a high-salt diet than animals on a low salt diet. A pathophysiological action of aldosterone involves development of extracellular matrix and fibrosis, inflammation, stimulation of reactive oxygen species production, endothelial dysfunction, cell growth and proliferation. Many studies showed local extra-adrenal production of aldosterone in brain blood vessel, and the heart, which contribute in an important manner to the pathological actions of this mineralocorticoid.Several studies such as RALES, EPHESUS, 4E and others, recently showed that mineralocorticoid-receptor (MR) antagonists, alone or in combination with ACE inhibitors or ARBs, reduced the risk of progressive target organ damage and hospitalization in patients with hypertension and heart failure. These clinical benefits support the therapeutic usefulness of MR antagonists.

## INTRODUCTION

Aldosterone is secreted by the zona glomerulosa of the adrenal gland in response to stimuli such as angiotensin II (Ang II), potassium, and adrenocorticotropic hormone[1]. The principal classical action of circulating aldosterone is the regulation of sodium and potassium transports in epithelial cells. To exert this and other actions, aldosterone binds to the cytoplasmic mineralocorticoid receptor (MR)[2]. The MR also binds cortisol and other glucocorticoid hormones with equal affinity as that for aldosterone. Tissue specificity for aldosterone is conferred by the local expression of the enzyme 11ß-hydroxysteroid dehydrogenase (11ßHSD) type 2, which converts cortisol and corticosterone into the inactive cortisone and 11-dehydrocorticosterone, respectively[3]. The binding of aldosterone to the MR results in the dissociation of the ligand-activated MR from a multiprotein complex containing molecular chaperones.

Translocation into the nucleus, binding to hormone response elements in the regulatory region of target gene promoters[4] induction of gene expression of serum and glucocorticoid inducible kinase-1 (sgk-1), Kirsten Ras (Ki.Ras A), and corticosteroid hormone-induced factor (CHIF), lead to the absorption of $Na^+$ ions and water through the epithelial sodium channel and potassium[5]. Numerous studies indicate that aldosterone not only contributes to salt and water homeostasis, but also exerts direct cardiovascular effects. In addition, many studies showed local extra-adrenal production of aldosterone in brain blood vessel and the heart[6,7,8]. The biosynthesis of aldosterone in extra-adrenal sites appears to be regulated by the same stimuli that regulate adrenal synthesis[8]. In the following pages, we will focus on the vascular, cardiac, and renal effects of aldosterone as mechanisms justifying the clinical benefits observed with the therapeutic use of MR antagonists.

## CARDIAC EFFECTS OF ALDOSTERONE: MECHANISMS INVOLVED

Numerous experimental and clinical studies indicate that aldosterone participates in cardiac alterations associated with hypertension, heart failure, diabetes, and other pathological entities. Circulating and locally produced aldosterone appears to be involved in the development of cardiac hypertrophy, fibrosis, and inflammation, these being the most remarkable heart alterations where this mineralocorticoid is involved[9,10,11]. Different mechanisms are involved in the above deleterious effects of aldosterone: increased cardiomyocyte calcium concentration, oxidative stress, inflammatory mediators, mitogen protein kinases, etc. It is important to mention that dietary salt is a key factor in aldosterone-mediated cardiac damage, since damage was more evident in animals on a high-salt diet than animals on a low-salt diet.

There is much evidence to indicate that aldosterone appears to be involved in myocardial fibrosis and remodeling in left-ventricular hypertrophy (LVH) in hypertensive states and during heart failure. Myocardial fibrosis has been observed in the hypertrophied left ventricle as well as in the nonhypertrophied right ventricle of rat[11] models of hypertension and hyperaldosteronism[10,11]. MR antagonists such as spironolactone or eplerenone prevented myocardial fibrosis in both ventricles, independently of the development of hypertension or LVH, indicating that aldosterone "per se" had important effects on structural alterations of the heart[12,13]. The mechanisms through which aldosterone causes cardiac hypertrophy and fibrosis have been widely investigated. Type 1 angiotensin II receptor (AT1) has been involved in the development of hypertrophy and fibrosis[14], and they have been found upregulated under these circumstances. Aldosterone increases AT1 density and mRNA in different cardiovascular tissues[15] and, consequently, it has also been proposed that the AT1 receptor would be a suitable mediator of cardiac fibrosis and hypertrophy. In fact, treatment with the AT1 receptor blocker losartan partially prevented aldosterone-induced cardiac hypertrophy and fibrosis in rats[15].

Many reports have indicated that intracellular calcium concentration plays an important role in the modulation of gene expression and growth in a variety of cell types, including cardiomyocytes. The importance of the calcium-dependent phosphatase, calcineurin, and its downstream transcription factor, NF-AT3, has been demonstrated[16] in the development of cardiac hypertrophy. In this sense, it has been shown that aldosterone is able to increase calcineurin activity and its mRNA expression in rat hearts, and a calcineurin inhibitor prevents aldosterone-induced cardiac hypertrophy independent of effects on blood pressure[17].

Aldosterone also exerts direct profibrotic effects. However, the salt content of the diet appears to be crucial for the profibrotic effect of aldosterone. In fact, infusion of aldosterone produces a dose-dependent increase in cardiac fibrosis in the presence of a high-salt diet, but not in the presence of a low-salt diet[11,15], suggesting that aldosterone-induced organ damage develops as a result of inappropriate plasma aldosterone levels for salt status. Uninephrectomized rats treated with aldosterone and drinking 1% saline for 8 weeks presented an elevation in blood pressure and cardiac fibrosis[9,18]. However, if rats were fed with a low-salt diet, neither blood pressure elevation nor cardiac fibrosis was observed, even in a state of hyperaldosteronism. This observation strongly suggests the possibility that excess of salt is a determinant factor for effects of aldosterone in nonepithelial tissues.

Recent studies proposed that the effect of aldosterone in inducing cardiac fibrosis is not directly exerted on cardiac myocytes or fibroblasts. Blood vessels have been proposed as the primary target organ of aldosterone in nonepithelial tissues. In fact, aldosterone produces functional and molecular alterations in the vessel wall, which could trigger subsequent mechanisms leading to myocardial fibrosis. Inflammatory cell infiltration and exaggerated expression of proinflammatory molecules, such as monocyte chemoattractant protein-1 (MCP-1), E and P selectines, and osteopontin, are increased prior to the development of fibrosis in animals treated with aldosterone and on a high-salt diet. Previous studies showed monocyte and macrophage infiltration, as well as fibrinoid necrosis around coronary arteries in an animal model receiving aldosterone with a high-salt diet. Furthermore, this effect was observed before the occurrence of cardiac fibrosis, and was accompanied by enhanced expression of inflammatory molecules, such as vascular cell adhesion molecule-1 (VCAM-1), cyclooxygenase-2 (COX-2), MCP-1, and osteopontin[19]. These molecular changes occurred earlier than structural alterations.

Other mechanisms apparently responsible for cardiac alterations induced by aldosterone involve the Na/H exchanger. The Na/H exchanger is important to maintain physiological pH in cardiovascular cells. Increased activity of the Na/H exchanger is involved in hypertrophy of cardiomyocytes in experimental myocardial infarction[20], spontaneously hypertensive rat (SHR)[21], and in mice with overexpression of β 1-adrenergic[22]. Inhibition of Na/H exchanger activity reduces cardiac hypertrophy. In patients with essential hypertension, the severity of cardiac hypertrophy seems to be correlated positively with activity of the Na/H exchanger[23]. Moreover, an increase in Na/H exchanger isoform 1 protein, and collagen deposition in perivascular and interstitial regions in the heart, were observed after 8 days of deoxycorticosterone administration in the presence of 0.9% saline[24]. The collagen deposition was inhibited by spironolactone and by the Na/H exchanger isoform 1 inhibitor, demonstrating the involvement of the Na/H exchanger in cardiac damage induced by mineralocorticoid and salt[24]. In addition, incubation of myocardial cells[25] and vascular smooth muscle cells[26] with aldosterone increased Na/H exchanger mRNA and activity. The inflammatory process seems also to be in this mechanism because increased expression of inflammatory molecules, inflammatory cell infiltration, and interstitial fibrosis were observed after administration of deoxycorticosterone with 0.9% saline to experimental animals. All the changes were found to be inhibited by administration of a Na/H exchanger antagonist[27].

## BLOCKADE OF MINERALOCORTICOID RECEPTORS: HUMAN STUDIES

Studies in patients with heart failure and hypertension support the mentioned experimental findings. In patients with heart failure, severity of LVH and myocardial fibrosis was associated with enhancement of myocardial expression of aldosterone synthase (CYP11B2), suggesting a role for locally synthesized aldosterone[28]. In large clinical trials in patients with left-ventricular dysfunction receiving ACE (angiotensin-converting enzyme) inhibitors, aldosterone production was correlated with mortality[29,30]. In addition, many clinical studies indicate a correlation between aldosterone concentrations and cardiovascular and renal morbidity and mortality. In a study evaluating the effects of circulating aldosterone on cardiac structural and functional changes, the response to salt-induced aldosterone suppression correlated with left-ventricular mass in patients with mild essential hypertension[31].

In the Randomized Aldactone Evaluation Study (RALES), in patients with heart failure (class 3 or 4 of New York Heart Association [NYHA]), the addition of spironolactone to the usual therapy (ACE inhibitor, diuretics, and digoxin) reduced mortality by 30%[12]. The specific mechanism underlying the beneficial effect of MR antagonism with spironolactone in RALES is not totally known. It has been proposed that spironolactone may prevent sudden death by increasing serum potassium, by altering myocardial norepinephrine uptake, or by reducing ventricular arrhythmias[31,32]. In addition, a substudy of RALES suggests that the antifibrotic effects of spironolactone could be related to the reduction in mortality[33]. In this study, spironolactone decreased circulating concentrations of the aminoterminal portion of the procollagen type II precursor, a marker of collagen turnover and predictor of mortality.

The Eplerenone Neurohormonal Efficacy and Survival Trial (EPHESUS) evaluated the consequences of the addition of the new MR antagonist eplerenone (25 to 50 mg/day) to standard therapy with ACE inhibitors, AT1 receptor antagonists, beta-blockers, digoxin, and diuretics on the primary end points of all-cause mortality and the time to first occurrence of either cardiovascular mortality or morbidity leading to hospitalization in 6200 patients with left-ventricular dysfunction (ejection fraction >40%) after a recent (3–14 days) myocardial infarction. The results indicated that addition of eplerenone significantly reduced all-cause and cardiovascular mortality[34].

The 4E Study (Eplerenone, Enalapril, and Eplerenone/Enalapril Combination Therapy in Patients with LVH) compared the effects of 9-month treatment with eplerenone 200 mg/day, enalapril 4 mg/day (n = 71), or eplerenone 200 mg/day plus enalapril 10 mg/day on left-ventricular mass, systolic and diastolic blood pressures, and urinary albumin-creatinine ratio (UACR) in patients with mild-to-moderate hypertension and echocardiographic evidence of LVH. Blood pressure reduction was similar among the three groups. All three treatments significantly reduced left-ventricular mass. The effect of combination enalapril and eplerenone on left-ventricular mass was significantly greater than the effect of eplerenone alone. Microalbuminuria was significantly reduced in the combination group compared with either the eplerenone or enalapril groups. These results suggest a favorable effect of eplerenone on microalbuminuria, an important risk factor for cardiovascular events[35].

## ALDOSTERONE IN RENAL DAMAGE: EXPERIMENTAL AND CLINICAL STUDIES

For years, the effect of aldosterone on salt and water homeostasis and potassium excretion acting through unidirectional transepithelial electrolyte transport was considered to be its predominant renal effect. However, it has been shown that aldosterone is an important factor involved in the progression of renal disease. One of first pieces of evidence supporting the deleterious effect of mineralocorticoids on kidneys showed that the administration of deoxycorticosterone acetate and salt in rats produced malignant hypertension associated with an important impairment of renal function[36]. These alterations were caused by the actions of mineralocorticoids and not the consequence of an activation of the renin-angiotensin system, because renin activity was very low and responded poorly to the administration of ACE inhibitors[37]. Hyperaldosteronism and adrenal hypertrophy are common features in experimental models of renal damage. In rats with remnant kidney, a tenfold increase in plasma aldosterone levels has been observed[38]. Ang II blockade clearly reduced hyperaldosteronism and attenuated proteinuria, hypertension, and glomerulosclerosis in this model[38]. Likewise, adrenalectomy also reduced the prevalence of hypertension, proteinuria, and structural renal alterations in these animals, demonstrating that the removal of the endogenous source of mineralocorticoids ameliorated the deleterious effects of renal ablation[39]. In stroke-prone SHR with high salt intake, a model of spontaneous hypertension that develops a malignant nephrosclerosis, the administration of the aldosterone receptor antagonists spironolactone or eplerenone markedly reduced proteinuria and renal damage. Similar results were observed when animals underwent adrenalectomy, supporting the participation of aldosterone in the renal damage associated with hypertension through mechanisms independent of hemodynamic effects[40]. The participation of aldosterone in renal damage has been shown in other models of hypertension such as rats fed a high-sodium diet and infused with both Ang II and NO synthesis inhibitor. Besides increase in blood pressure, the animals develop proteinuria as well as fibrinoid necrosis of renal arterioles[41]. Renal damage was inhibited by the administration of eplerenone or the total adrenolectomy, supporting the role of aldosterone in the renal damage induced by Ang II/salt This participation was further supported by the fact that administration of aldosterone in adrenolectomized animals reproduced the renal damage observed in intact rats[42].

Numerous clinical studies showed an association between high levels of aldosterone and renal deterioration. In an early study, a higher incidence of high aldosterone plasma levels in patients with renal failure was found despite normal serum potassium levels and plasma renin activity[43,44]. This relationship is also confirmed by the fact that the majority of the patients with primary aldosteronism present proteinuria[45]. In the last years, several clinical studies have supported the participation of aldosterone in renal damage associated with diabetes and hypertension since antagonists of MRs show a renoprotective effect in these pathological situations. The administration of spironolactone in patients with chronic renal disease who received ACE inhibitors further reduced the protein excretion induced by blocking the renin-angiotensin system[46]. Similarly, the addition of spironolactone in patients with diabetes type 2 and early nephropathy treated with enalapril reduced microalbuminuria without changes in blood pressure. These results suggest that aldosterone blockade can exert a beneficial effect in a diabetic patient treated with ACE inhibitors by reducing not only renal, but also cardiac, damage since it was also able to reduce left-ventricular mass index[47]. In patients with diabetes type 2 and mild-to-moderate hypertension, the combination of eplerenone with enalapril was more effective in reducing proteinuria after 24 weeks of treatment than any individual treatment. This additional antiproteinuric effect induced by eplerenone was independent of blood pressure lowering[48]. A similar situation was observed in patients with essential hypertension with LVH that were treated with either eplerenone, enalapril, or the combination[35]. Since there was a poor correlation between the beneficial effects of eplerenone and changes in blood pressure, the results suggested that the deleterious effects of aldosterone could not be simply explained by changes in blood pressure. Supporting these results was the observation, in elderly patients with systolic hypertension, that eplerenone reduced the urinary albumin/creatinine ratio to a larger extent than did amlodipine, besides the similar reduction in blood pressure induced by both treatments[49].

## MECHANISMS INVOLVED IN RENAL DAMAGE INDUCED BY ALDOSTERONE

The mechanisms underlying the deleterious effect of aldosterone in renal damage could involve the activation of different molecular pathways that can also participate in the organ damage induced by aldosterone in other tissues. Besides its participation in fibrinolisis, plasminogen activator inhibitor 1 (PAI-1) is a profibrotic factor through its ability to modulate extracellular matrix and the stimulation of growth factors[50]. It has been shown that PAI-1 can participate in the development of glomerulosclerosis and tubulointerstitial fibrosis[51]. Since levels of PAI-1 correlate with aldosterone concentrations during periods of low salt intake[52], it has been suggested that PAI-1 could participate in the injury and fibrosis induced by aldosterone. This affirmation is based on the observation that in rats undergoing kidney radiation, aldosterone blockade reduced the development of proteinuria and glomerulosclerosis observed in these animals. This beneficial effect was accompanied by a reduction in PAI-1 expression[53], supporting the idea that the profibrotic effects of aldosterone could involve stimulation of this factor. In addition, TGF-β can be another mediator of the profibrotic effect of aldosterone. TGF-β is a cytokine that can stimulate differentiation and proliferation of fibroblasts and collagen deposition. In uninephrectomized rats, aldosterone infusion was associated with an increase in TGF-β and collagen expression that was accompanied by medullary and cortical fibrosis. All these effects were observed in the presence of an AT1 receptor antagonist, suggesting that the effect of aldosterone is independent of Ang II actions[54].

As already mentioned for the heart, inflammation can be one important mechanism involved in the renal injury induced by aldosterone. In aldosterone/salt hypertension, albuminuria and renal vascular injury were accompanied by an important renal vascular inflammatory process characterized by an increase in several cytokines such osteopontin, interleukin 1-β, interleukin-6, and MCP-1. The administration of the aldosterone receptor antagonist, eplerenone, reduced vascular, glomerular, and tubular damage as well as the inflammatory process[55]. This inflammatory response induced by aldosterone in the kidney could be mediated by the activation of transcription factors NFκB and AP-1, which are involved in the stimulation of different inflammatory markers. In transgenic rats overexpressing both human renin and angiotensinogen genes (dTGR), eplerenone reduced the renal activity of these transcription factors and leukocyte infiltration[56]. In aldosterone/salt-hypertensive rats, renal damage was accompanied by an increase in oxidative stress in the renal cortex, suggesting that changes in redox status are involved in the progression of renal injury induced by aldosterone[57]. This participation was confirmed by the fact that treatment with the antioxidant tempol normalized reactive oxygen species (ROS) levels and prevented the development of renal damage in these animals. Similarly, ROS have been implicated as mediators of renal injury in different models of hypertension, including SHR, DOCA-salt hypertension, and Dahl salt-sensitive hypertensive rats[58,59,60], in which the role of aldosterone has been implicated in the glomerulosclerosis observed in these models. This increase in ROS induced by aldosterone seems to involve the stimulation of NADPH oxidase, the main enzyme involved in the production of superoxide anions because aldosterone/salt rats presented an increase in mRNA expression of different subunits of this enzyme in the renal cortex. This increase has also been reported in other organs, such as vascular wall[61]. Since it has been shown that ROS stimulate different intracellular pathways involved in several processes such fibrosis and inflammation, it is possible to suggest that oxidative stress plays a key role in aldosterone-mediated renal damage.

## VASCULAR EFFECTS OF ALDOSTERONE

In addition to adrenal medulla, vascular smooth muscle and endothelial cells are able to synthesize aldosterone[62]. This synthesis is mainly regulated by Ang II and potassium at a transcriptional level[64]. Experimental and clinical evidence indicates that aldosterone, acting on smooth muscle and endothelial cells, induces vascular alterations through endocrine and/or paracrine mechanisms[63]. These alterations affect vasoreactivity, oxidative status[41,57,65], endothelial function, and produce inflammation, stiffness, and fibrosis[19,66]. Different mechanisms have been implicated in these deleterious effects: magnesium deficiency, increased intracellular calcium concentration, increase of ROS, overexpression of inflammatory mediators, mitogen-activated protein kinases, etc.[66,67,68,69]. Additionally, recent studies have reported that dietary salt can modify aldosterone-mediated vascular injury, since vascular damage was greater in animals on a high-salt diet than in animals on a low-salt diet[70,71]. Aldosterone receptor antagonist attenuates aldosterone-mediated vascular injury by mechanisms that appear to be independent of changes in blood pressure[72,73].

### Endothelial Function and Vascular Reactivity

The endothelium plays a role in the control of vascular tone by releasing different factors including NO and prostanoids. A growing number of studies indicate that aldosterone contributes to endothelial dysfunction, which is characterized by reduced endothelium-dependent relaxations[74]. Systemic aldosterone infusion in healthy young volunteers induces endothelial dysfunction, which is mediated by MR activation[75]. This effect may be the result of a reduction in NO release and/or an increase in NO inactivation. An increase in NO metabolism is associated with an increase in superoxide anions, which scavenge NO to form peroxynitrite[76]. Peroxynitrite is a highly reactive oxidizing species whose cytotoxic effect induces depolarization of the mitochondrial membrane, activates both caspase-9 and caspase-8, and inactivates ATP synthetase, aconitase, and creatine kinase[77]. Reactive ROS production has been involved in the deleterious effects of aldosterone on vascular beds. Vascular NADPH oxidase activity and ROS production are increased by aldosterone treatment in several experimental situations[67]. Endogenous aldosterone participates in the vascular alterations associated with hypertension in rats. We recently reported that treatment of SHR with eplerenone enhanced acetylcholine-induced relaxations. This effect of eplerenone was associated with increased aortic expression of eNOS mRNA expression and a reduction of NADPH oxidase mRNA expression[65]. These results suggested that endogenous aldosterone is related to endothelial dysfunction and a diminished NO availability by increasing oxidative stress in SHR[65] .

An alternative general mechanism leading to endothelial dysfunction is related to the exaggerated production of vasoconstrictor prostanoids. We recently found that chronic treatment of normotensive and hypertensive rats with aldosterone produced endothelial dysfunction. This effect was associated with COX-2 activation. Furthermore, overproduction of prostacyclin, which could also act as a vasoconstrictor factor, was involved in this process. Thus, it appears that aldosterone is able to stimulate COX-2 and prostacyclin production[73].

One of the earliest identified effects of aldosterone was its capacity to modulate vascular smooth muscle tone, through the modification of both vasoconstrictor and vasodilator responses. Two mechanisms have been associated with changes in vascular tone. A genomic mechanism, acting through protein synthesis, and a nongenomic mechanism that modulates intracellular calcium, cAMP levels, Na+/H+ exchanger activity, and phosphorylation of signaling molecules[19,78,79,80]. The effects of aldosterone in the vascular wall includes direct induced contractions and enhancement of sensitivity to vasoconstrictors[78,81,82]. Vasoconstriction in rat mesenteric arteries was abolished by PKC, Na+/H+ exchanger-1 inhibitor, and by eplerenone[83]. In rabbit afferent arteriole, NO modulates the vasoconstrictor action of aldosterone, which in turn is mediated by IP3 and PKC[84]. The mechanism underlying the contraction induced by aldosterone has been associated with catecholamines, either through an increased release or a decreased reuptake. Increased intracellular pH and calcium levels, as well as an upregulation of Ang II receptors, have also been proposed as mechanisms involved in the constrictor action of aldosterone[83,85,86,87].

Finally, it is important to mention that aldosterone could increase NO release due to NO synthase activation. This effect has been reported to decrease the vasoconstrictor response in rabbit preglomerular afferent arterioles[88]. β-Adrenoceptor upregulation has also been associated with vasodilation[89]. We recently reported that aldosterone decreased the vasoconstrictor response to electrical field stimulation in mesenteric arteries from SHR, through an increased vasodilator response to sensory neurotransmitter calcitonin-gene-related peptide[90]. Collectively, the results indicate that aldosterone is an important factor in the modulation of vasomotor toner through multiple mechanisms, which vary with species and the vascular bed studied.

## Inflammation

Aldosterone has been related to vascular inflammation. Different rat models (NO inhibition plus high Ang II, high-salt diet in presence of Ang II, aldosterone-infused uninephrectomized rat, stroke-phone SHR, etc.) have been used to explore the inflammatory effect of aldosterone[19,55,91,92]. All these studies showed that aldosterone is able to induce vascular expression of inflammatory mediators. Recent studies from our laboratory suggested that endogenous aldosterone participates in the vascular inflammatory process associated with hypertension in the SHR. In this study, treatment with eplerenone reduced enhanced vascular expression of cytokines through the modification of NFKb/IkB system[92]. As mentioned above, ROS production plays a crucial role in the deleterious effects of aldosterone on vascular wall. Aldosterone-stimulated production of superoxide anions is a feasible mechanism able to activate NFkB, and the genes regulated by this transcription factor[65,63,93]. Other inflammatory mediators have also been involved in the effects of aldosterone. Aldosterone administration in rats increases coronary expression of COX-2, osteopontin, and macrophage chemoattractant protein-1, supporting the notion that this mineralocorticoid is importantly involved in vascular proinflammatory process[19,42]. Finally, it has been recently suggested that the inflammatory response to aldosterone could be mediated, in part, by a fall in magnesium content and calcium loading of peripheral blood mononuclear cells. This effect was associated with an enhancement of H2O2 production[69].

## Structural Changes

Besides functional effects, chronic administration of aldosterone was able to induce structural vascular alterations, including hypertrophy, remodeling, stiffness, and fibrosis[93,94,95]. It has been shown that aldosterone increases incorporation of tritiaded leucine into vascular smooth muscle cells in presence of a low dose of Ang II. This effect was inhibited by specific aldosterone antagonism, indicating that aldosterone directly induces hypertrophic changes in vascular smooth muscle cells[94]. The mechanisms underlying the structural changes produced by aldosterone are unclear. Mediators, such as Ang II, endothelin-1, ROS, and high salt intake seem to play an important role in the structural vascular alterations produced by aldosterone under different pathological and experimental conditions[41,65,67,71,95].

Most of the studies on aldosterone and development of fibrosis focused preferentially on the heart, with only a few recent reports focused on vascular fibrosis. These studies reported that the mechanisms involved in aldosterone induction of vascular fibrosis are similar in vessels and in the heart. Vascular fibrosis involves the accumulation of extracellular matrix protein such as collagen, elastin, fibrillin, fibronectin, and proteoglycans in the vascular media. The mechanisms involved in vascular fibrosis, as mentioned for smooth muscle cell hypertrophy, seem to be mediated by increased ROS and endothelin-1 production, as well as salt loading[67,93,95]. However, it has recently been demonstrated that the fibrotic effect of aldosterone is salt loading–independent in resistance arteries[67]. In both situations, with and without high salt intake, the increased oxidative stress seems to be the main mechanism implicated[65,67]. We recently demonstrated that endogenous aldosterone participates in the structural alterations observed in SHR. This effect was associated with an enhanced expression of NADPH oxidase, suggesting a potential production of ROS. As mentioned above, this was not associated with variations in salt intake[65].

## POTENTIAL ADVERSE EFFECTS OF MR ANTAGONISTS

In the majority of the clinical trials, the adverse effect profile of MR antagonists, given alone or in combination with other antihypertensive medications, was not significantly different from that of placebo, with the exception of the increased risk of hyperkalemia[96]. However, it should be emphasized that the risk of hyperkalemia increases during combination therapy with ACE inhibitors or Ang II receptor blockers (ARBs). In the EPHESUS study, the incidence of serious hyperkalemia, defined as a serum potassium concentration >6 mEq/l, was 5.5% in the eplerenone group and 3.9% in the placebo group. Incidence of hyperkalemia with eplerenone seems to be similar to that seen with spironolactone[34]. In the RALES study, the incidence of serious hyperkalemia increased with increasing dosages of spironolactone, from 5% with 12.5 mg/day to 13% with 25 mg/day, 20% with 50 mg/day, and 24% with 75 mg/day[12]. Diabetic microalbuminuria seems to be a specific area for concern because the incidence of hyperkalemia was higher in this specific type of patient. However, eplerenone was particularly efficacious in this subgroup in terms of reducing microalbuminuria. However, in this subgroup of patients, the greater efficacy of eplerenone was indeed matched by a greater incidence of the adverse effect of hyperkalemia. This is a good example of how, in clinical therapeutics, benefit and risk sometimes go together. Thus a careful monitoring schedule in hypertensive diabetic patients should be done to identify those individuals prone to develop hyperkalemia.

Finally, another important aspect of aldosterone antagonists is the rate of sex hormone–related adverse events, which have been much lower with eplerenone than with spironolactone. The incidence of gynecomastia or breast pain was significantly greater in men receiving spironolactone compared with those receiving placebo (10 vs. 1%, respectively; $p \leq 0.001$). In eplerenone-based studies, sex hormone–related events have been reported to be comparable to that seen with placebo.

## CONCLUSION

Aldosterone is an important agent and mediator of cardiovascular and renal injury in a number of experimental models and human diseases. Numerous studies have shown that aldosterone/salt imbalance is detrimental to patients with hypertension and heart failure, and can lead to progressive damage in the heart, vasculature, and kidneys. Moreover, much evidence suggests that aldosterone enhances tissue-damaging effects of Ang II and other agents. For years, ACE inhibitors and ARBs have been used in hypertension, heart failure, kidney disease, etc. and the participation of aldosterone in the pathophysiological mechanisms underlying these entities was ignored. Although ACE inhibitors and ARBs reduce plasma aldosterone initially, aldosterone levels rebound, or escape, during long-term therapy. Consequently, aldosterone again becomes available to continue exacerbating cardiovascular damage. Therefore, the blockade of aldosterone, alone or in combination with ACE inhibitors or ARBs, reduces the risk of progressive target organ damage in patients with hypertension and heart failure.
